# Outcomes of Crowding in Emergency Departments; a Systematic Review

**Published:** 2019-08-28

**Authors:** Hamid Reza Rasouli, Ali Aliakbar Esfahani, Mohammad Nobakht, Mohsen Eskandari, Sardollah Mahmoodi, Hassan Goodarzi, Mohsen Abbasi Farajzadeh

**Affiliations:** 1 Trauma Research Center, Baqiyatallah University of Medical Sciences, Tehran, Iran.; 2 Marine Medicine Research Center, Baqiyatallah University of Medical Sciences, Tehran, Iran.

**Keywords:** Crowding, outcome assessment, emergency service, hospital, systematic review

## Abstract

**Introduction:**

Emergency Department (ED) crowding is a global public health phenomenon affecting access and quality of care. In this study, we seek to conduct a systematic review concerning the challenges and outcomes of ED crowding.

**Methods:**

This systematic review utilized original research articles published from 1st January 2007, to 1st January 2019. Relevant articles from the PubMed (MEDLINE), EMBASE, and Google scholar databases were extracted using predesigned keywords. Following the PRISMA guidelines, two reviewers independently evaluated the quality of the studies using Critical Appraisal Skills Programme for cohort studies and qualitative studies, and Joanna Briggs Institute Meta-Analysis of Statistics Assessment and Review Instrument for studies.

**Results:**

Out of the total of 73 articles in the final record, we excluded 15 of them because of poor quality. This systematic review synthesized the reports of 58 original articles. The outcomes of multiple individual patients and healthcare-related challenges are comprehensively assessed.

**Conclusions:**

ED crowding affects individual patients, healthcare systems and communities at large. The negative influences of crowding on healthcare service delivery result in delayed service delivery, poor quality care, and inefficiency; all negatively affecting the emergency patients' healthcare outcomes, in turn.

## Introduction

The requirement of emergency healthcare service is an ongoing issue (1). The emergency department (ED) is expected not only to provide emergency care to patients but also to fulfill the needs of the providers, and the communities at large. Besides, the emergency department might be the only source of healthcare services to people especially in rural communities (1, 2). 

Evidence shows an increase in emergency healthcare service utilization because of the increased rates of accidental injuries. However, the capacity of the emergency healthcare systems has not been well developed to respond to such high demand because creating a balance between emergency services and the required resources is challenging, especially in under-resourced countries (3-5). This condition leads to crowding of the EDs, which in turn impose public health challenges related to quality of healthcare and outcomes. Crowding is a situation when an identified need for emergency healthcare services exceeds the available resources to provide emergency care to patients within an appropriate time frame (1, 3, 6). 

Crowding of the ED leads to adverse outcomes for the patients, providers, the healthcare system and the community. Delay in service provision to patients not only can compromise the quality of the emergency services but can also worsen their consequences. Crowding of the ED might also lead to the violations of the norms and the service provision standards, which in turn might result in patients leaving the facilities without getting the required services. Thus, this systematic review aims to describe the consequences of ED crowding for emergency patients, emergency care providers, and healthcare systems. The findings are anticipated to provide inputs to decision-makers for a better understanding of the effects of ED crowding and to contextualize practical solutions to improve the quality of medical emergency services.

## Methods

**Search Strategy**

In this review, we adopted the definition for “crowding” from the American College of Emergency Physicians which states “Crowding occurs when the identified need for emergency services exceeds available resources for patient care in the emergency department, hospital, or both.” Then, we searched for articles related to crowding in EDs and its major outcomes published in English between January 1, 2007, and January 1, 2019, in PubMed (MEDLINE) and Embase electronic databases. We applied search terms based on common keywords in the literature concerning the consequences of emergency department crowding (Table 1). We used suitable combinations of "OR" and "AND" in all databases. Also, we searched Google scholar and Google to find relevant papers. 

**Data collection and quality assessment**

Two reviewers (HR.R. & A.AE.), independently screened the titles, abstracts and the methodological validity of the records using data extraction format before their inclusion in the final review. Discussions with the senior author (M.E) were used to resolve any disagreements among the reviewers during the assessment phase. 

The inclusion criterion was: All studies evaluating the effects and consequences of ED crowding. However, a study was excluded if it only reported the outcomes of a case report or systematic review investigations.

**Figure 1:**
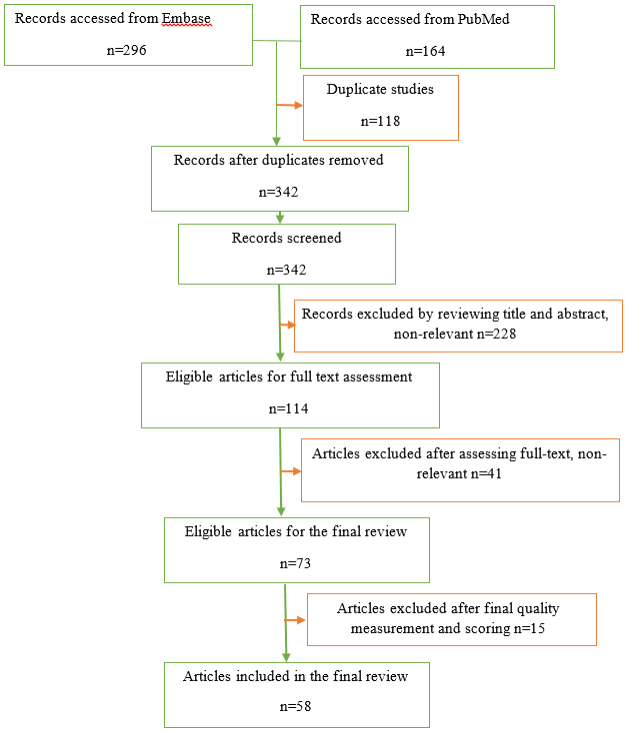
Study selection flowchart

A total of 73 articles were eligible for the review (Figure 1). We further assessed the records using the standardized Critical Appraisal Skills Programme (CASP) for the Cohort Studies, and Qualitative Studies. Besides, the Joanna Briggs Institute Meta-Analysis of Statistics Assessment and Review Instrument (JBI-MAStARI) for studies which employed other designs was used (7). We addressed PRISMA checklist requirements. Finally, after excluding 15 records with eligibility assessment scores below 0.33 points (<33%), the final review was done on 59 records. Throughout the processes, we attempted to maintain the original intentions of authors such as effects on patients, effects on healthcare delivery process, effects on quality care, and effects on efficiency in service delivery.

**Ethics approval and consent:** The research protocol was approved by the review committee of the Baqiyatallah University of Medical Sciences.

## Results

Our search initially retrieved 158 studies. However, 132 papers were excluded by reviewing title and abstract and assessing full-text due to non-relevance. Then, 15 studies were excluded after final quality measurement and scoring for primary screening due to receiving below 0.33 points (<33%). Finally, 58 eligible peer-reviewed original articles were included in the final review (Figure 1 and Table 2).

The consequences of patient crowding in hospitals are multifaceted involving effects related to patient health outcomes, healthcare delivery system and the community at large. Table 3 presents a summary of the commonly reported outcomes of ED crowding. ED crowding leads to delayed care for emergency patients and risk of not being visited by clinical care providers in a timely manner (8-14). The patients may react to prolonged stay to get services and to the crowding by frequent walkouts (15). The worsening of their illness (16) could result in frequent re-admissions (17, 18), prolonged hospitalizations (16, 19, 20), and related costs (21). Dissatisfaction of emergency patients (22-25), medication errors and adverse events (26-29), and patient death (16, 17, 19-21, 30-36) were also common consequences. 

The response to emergency and non-emergency patients influences the quality of services provided, patients’ outcomes and the healthcare system. Discharge of patients even with high-risk clinical features (17) and diverting the patients to other facilities (37) might have affected the health outcomes. These conditions not only decrease admission rates (38) and prolong the time to receive and transfer outpatients (39), but also compromise the patients' health outcomes and lead to high admission and re-admission rates (22, 36, 40) followed by a decrease in discharge rate of patients (17). In addition, the prolonged hospitalization of patients leads to overutilization of diagnostic and other laboratory facilities (40). 

The crowding of the EDs negatively influences both the healthcare delivery process and the outcomes. The high workload (41) results in delayed service provision, delayed clinical decision making, and increased length of stay (LOS) of patients (20, 21, 31, 35, 36, 40-54). These situations negatively influence the quality of services and efficiency (8, 14, 16, 36, 55-58). A properly managed medical emergency contributes to the prevention of the event in communities. For example, a successfully treated patient with community-acquired pneumonia will be less likely to transmit the disease to other community members (13). 

**Table 1:** Keywords used for searching published articles in databases

	**Emergency department related concepts**		**Crowding related concepts**		**Outcome related concepts**
	**Controlled phrases **	**Keywords**	**Controlled term/phrase**	**Keywords**	**Keywords**
**PubMed**	emergency medicine, pediatric emergency medicine, hospital emergency service, emergency medical services	emergency, emergency medicine, pediatric emergency medicine, emergency medical services, emergency room, hospital emergency services, emergency health services, emergency department, emergency ward, ER, ED	crowding	crowding, overcrowded, crowded overcrowding, divert, diversion, congestion, surged, surging, capacity, crises, crisis, occupancy, hospital bed utilization, bed, utilization	Left without being seen (LWBS), Length of stay (LOS), delayed treatment, satisfaction, adverse events mortality, morbidity, error, hospitalization, quality, performance, readmissions, overutilization, efficiency, cost
**EMBASE**	emergency ward, emergency medicine		crowding, hospital bed utilization		

**Table 2:** Studies examining outcomes of emergency department (ED) crowding

**Author** ** Year**	**Study Design**	**Sample**	**Quality**	**Outcome variable**
Cremonesi,2015	survey	54,254 patients	High	average per-patient cost; severity of health condition
Wang,2015	prospective pilot	3139 patients	High	average length of stay (LOS); patient Left without being seen (LWBS)
Shenoi,2009	cross-sectional	63,780 admissions	High	diversion
Fee,2007	cross-sectional	39,000 visits	High	ED volume at the time of arrival
Ben-Yakov,2015	cohort	9,759 patients	High	ED crowding; patient disposition (admission/discharge)
Cha,2011	regression	125,031 patients	High	mean patient volume over 8-hour; hospital mortality
Chang,2017	longitudinal	2,619 hospitals	High	LOS for admitted patients
Chiu,2017	cohort	70,222 visits	High	ED occupancy status; decision-making time; LOS; patient disposition
Depinet,2014	cross-sectional	9,976 patients	High	time to critically abnormal vital sign reassessment; patients waiting for admission, patients waiting in the lobby
Derose,2014	cohort	136,740 patients	High	inpatient mortality; ED LOS
Dubin,2013	retrospective	69 patients	High	emergency physician (EP) errors; number of patients boarding at the time of patient disposition
Epstein,2012	cohort	533 patients	High	occurrence of preventable medical errors; ED Occupancy
Fee,2011	cross-sectional	486 patients	High	arrival-to-antibiotic-administration times; number of ED patients requiring admission at the time of arrival
Gabayan,2015	cohort	625,096 visits	High	inpatient admission; death within 7 days
Gaieski,2017	cohort	2913 patients	High	ED occupancy; waiting patients; time to antibiotics; mortality
Hong,2013	cross-sectional	1296 patients	High	delayed resuscitation efforts; hospital mortality
Hsia,2013	cross-sectional	3,368,527 patients	High	ED crowding; bounceback admission
Hwang,2008	cross-sectional	1,068 patient	High	number of admitted patients; pain care measures
Jo,2012	cross-sectional	477 cases	High	28-day mortality; timeliness of antibiotic therapy
Jo,2014	cross-sectional	54,410 patients	High	Emergency department occupancy ratio; ED LOS
Jo,2015	cross-sectional	1801 patients	High	ED occupancy ratio; inpatient mortality
Kennebeck,2011	cohort	190 patients	High	ED crowding; timeliness of antibiotic administration
Kulstad,2009	cross-sectional	17 patients	High	time to the first electrocardiogram (ECG); time to patient arrival in catheterization laboratory; occupancy rate
Kulstad,2010	observational	NA	High	average daily occupancy rate and the emergency department work index (EDWIN) score; number of medication errors
Lee,2012	prospective review	11491 adults	High	ED crowding
McCarthy,2009	cohort	4 EDs	High	crowding at 30-minute intervals throughout each patient's ED stay; waiting room time; treatment time; and boarding time; occupancy rate
McCusker,2014	cohort	677,475 patients	High	30-day outcomes: mortality, return ED visits, occupancy ratio separately for ED bed and waiting room patients
Medley,2010	prospective review	6,640 imaging studies	High	number of radiology studies ordered per patient; occupancy rate
Michelson,2012	cohort	198,778 visits	High	ED occupancy rate; return visits to the ED within 48 hours
Mills,2009	cross-sectional	976 patients	High	administration of and delays in time to analgesia
Mills,2010	prospective cohort	767 patients	High	ED crowding; time from triage to computed tomography (CT) read
Muller,2015	cross-sectional	40 ED bed	High	time to initial physician assessment; and daily nursing hours
Mullins,2014	ecological	4810 hospitals	High	LWBS; waiting times; boarding times; and LOS for admitted and discharged patients
O'Connor,2014	pilot	500 patients	High	triage time; date; treatment area; time to physician initial assessment; return ED visits within 14 days
Pines,2007	cohort	694 patients	High	delay (>4 hours from arrival)
Pines,2007	cross-sectional	741 patients	High	ED crowding
Pines,2008	cohort	1,469 patients	High	ED crowding (hallway placement, waiting times, and boarding times); patient satisfaction
Pines,2008	cohort	13,758 patients	High	Poor care; a delay (>1 hour) from triage to first pain medication; a delay (>1 hour) from room placement to first pain medication
Pines,2009	cross-sectional	4574 patients	High	inpatient adverse outcomes
Pines,2010	retrospective cohort	1,716 patients	High	ED crowding; ED occupancy, waiting patients, admitted patients, and patient-hours); overall LOS; time to treatment
Reznek,2017	retrospective	463 patients	High	Door-to-Imaging Time (DIT) within the 25-minute goal
Shenoi,2011	cross-sectional	161 patients	High	ED census; time to analgesic administration
Shin,2013	retrospective	770 patients	High	ED occupancy rate; compliance
Sikka,2010	correlation	334 patients	High	overall time to antibiotic administration
Sills,2011	cross-sectional	927 patients	High	ED occupancy; number waiting to see an attending-level physician
Sun,2013	cohort	995,379 ED visits, 187 hospitals	High	inpatient mortality; hospital length of stay; costs
Tekwani,2013	cross-sectional	1591 surveys	High	ED crowding; hospital diversion status; satisfaction
van der Linden,2014	cohort	169 patients	High	walkout from emergency
Van Der Linden,2016	retrospective	39110 patient	High	time to triage; time to treatment; age; 24-h mortality; 10-day mortality.
van der Linden,2016	cross-sectional	49539 patient	High	occupancy ratio; ED occupancy; LOS; time to triage
Verelst,2015	cohort	108,229 patients	High	in-hospital death; hospital; acquired morbidities; total hospital stay
Wang,2017	cohort	1345 participants	High	ED crowding; patient real-time satisfaction.
Ward,2015	cross-sectional	405 hospitals	High	admitted LOS; discharged LOS; boarding time; waiting time
Wiler,2013	cross-sectional	87,705 visits	High	patient LWBS
Wu,2015	cohort	852 patients	High	inpatient outcomes
Phillips, 2017	cohort	2,557 patients	High	ED LOS
Higginson, 2017	cross-sectional	NA	High	bed occupancy
Geelhoed,2012	quasi-experimental	NA	High	mortality rates; overcrowding rates

**Table 3:** Effects of crowding in emergency departments

**Effects on patients**
Delayed assessment or treatment; not being seen; not given care (8-14) Increased walkouts due to perceived ED length of stay (LOS) (15) Morbidity (16) Frequent readmissions (17, 18) Prolonged hospitalization (16, 19, 20) The high cost of treatment (21) Low satisfaction (22-25) Medication errors and adverse events (26-29) Mortality (16, 17, 19-21, 30-36)
**Healthcare delivery system process**
High workload (41) Delayed service provision/decision making and increased ED LOS (20, 21, 31, 35, 36, 40-54) Discharging patients with high-risk clinical features (17) Diverting patients to other facilities to reduce load (37) High patient re-admission rate (22) Decreased admission of patients due to crowding (38) Decreased discharge rate of patients despite crowding (17) High patient admission rate to general wards and ICU (40) Overutilization of diagnostic imaging and laboratory tests (40) Prolonged time to receive and transfer outpatients (39)
***Effects on quality care***
Shorter time to investigate patients’ conditions (49) Poor infection prevention and control measures (63) Low compliance with standards of care (19) Compromised quality of care (12, 22, 41, 51, 57, 64-66) High bed occupancy rate
***Effects on efficiency in service delivery***
Poor performance, low efficiency, and high cost of care/treatment (8, 14, 16, 36, 55, 56, 58)

## Discussion

This systematic review synthesized the outcomes related to ED crowding in hospitals. Crowding of ED can result in consequences for emergency patients’ health outcomes, the healthcare delivery system, and the community at large. 

The high inflow of emergency patients to ED leads to crowding of the ED, which can in turn negatively affect the healthcare delivery process and outcomes. Delayed emergency healthcare service provision and patients leaving without being seen (LWBS) (8-14) have been commonly identified as consequences of crowding. This condition could inevitably lead to increased walkout of patients due to the perceived high length of stay. As a result, the emergency patients' morbidity worsened, and subsequent mortalities increased (16, 17, 19-21, 30-36). The frequent readmissions and prolonged hospitalizations of emergency patients not only increase ED crowding, but also negatively affect the cost of treatment (21) and patient satisfaction (22-25). Hoot and Aronsky in their systematic review identified a direct relationship between ED crowding and emergency patient death, reduced quality of care, and increased treatment costs (59). Delayed patient assessment and care provision could result in increased mortality, medical error, and decreased patient satisfaction (60).

The increase in the workload of emergency healthcare staff due to the high patient flow results in delayed clinical decision making and emergency healthcare service provision and increased ED LOS of patients (20, 21, 31, 35, 36, 40-42, 44-54, 61, 62). This condition again leads to discharge of patients even with high-risk clinical features (17) and to the diversion of emergency patients to other health facilities (37). ED crowding can also be associated with decreased admission rates (38), delayed emergency healthcare provision, and delay in transfer of emergency patients to inpatient wards (39). In contrast, the high admission and re-admission rates of emergency patients (22, 36, 40) followed by a decreased patient discharge rates (17) and prolonged hospitalization can lead to overutilization of diagnostic imaging and laboratory tests (40). Thus, several emergency healthcare-related consequences seem to be overlooked in the Morley et al. synthesis as they mainly focused on inpatient LOS and ED LOS (60). Our review broadly highlighted the healthcare delivery system-related consequences of ED crowding under the categories of healthcare delivery process, quality care, and efficiency. 

ED crowding can negatively affect the quality of emergency healthcare. The higher the number of emergency patients, the longer the time it takes to investigate their conditions and to take supportive actions (49). These conditions can lead to reduced emergency healthcare quality and poor healthcare outcomes, which may result in an increase in bed occupancy rate (63). Besides, these conditions may negatively affect performances and result in inefficiency due to an increase in treatment costs (8, 14, 16, 36, 55, 56, 58). Similarly, others also identified the negative influence of ED crowding on the cost of treatment (59) and non-adherence to best practice guidelines for emergency service provision (60).

**Strengths and Limitation**

This systematic review synthesized original articles related to outcomes of the emergency department crowding in hospitals globally. Several studies identified complex issues related to emergency department crowding. Our review identified several crowding-related challenges and consequences including patient and staff reactions. The relevant original articles on ED crowding were accessed from the PubMed, Embase, and google scholar databases using comprehensive search keywords. The qualities of the records have been assessed using relevant checklists and those with low quality have been excluded. Our review also adds to the comprehensiveness of the view about the issues. The more explicit schematization of our synthesis compared to other existing reviews can facilitate a better understanding of the complex phenomenon. However, this review has certain limitations. It used study reports published only in English retrieved from the two mentioned sources. Moreover, the reviewed studies did not have a shared definition of crowding.

## Conclusion:

ED crowding affects individual patients, healthcare systems and communities at large. The negative influences of crowding on healthcare service delivery result in delayed service delivery, poor quality care, and inefficiency; all negatively affecting the emergency patients' healthcare outcomes, in turn. This review highlights the importance of response to emergencies and emergency-related crowding and preventing the consequences to better address the healthcare needs of emergency patients and increase the effectiveness of healthcare service delivery centers.

## List of abbreviations

ED: Emergency Department

MeSH: Medical Subject Headings 

CASP: Critical Appraisal Skills Programme

JBI-MAStARI: Joanna Briggs Institute Meta-Analysis of Statistics Assessment and Review Instrument

LWBS: Left Without Being Seen

LOS: Length of Stay

## Availability of data and supporting materials:

The datasets used and analyzed during the current study are available from the corresponding author on request.

## Conflicts of interests:

No conflicts of interest

## Funding:

Not applicable

## Authors' contributions:

All the authors have contributed to development of the concept and production of the final manuscript.
